# Sodium *p*-Toluenesulfinate Enhances the Bonding Durability of Universal Adhesives on Deproteinized Eroded Dentin

**DOI:** 10.3390/polym13223901

**Published:** 2021-11-11

**Authors:** Yorichika Shioya, Antonin Tichy, Kazuhide Yonekura, Mayu Hasegawa, Takashi Hatayama, Masaomi Ikeda, Junji Tagami, Masatoshi Nakajima, Keiichi Hosaka

**Affiliations:** 1 Department of Cariology and Operative Dentistry, Graduate School of Medical and Dental Sciences, Tokyo Medical and Dental University, 1-5-45 Yushima, Bunkyoku, Tokyo 113-8510, Japan; shioya.ope@tmd.ac.jp (Y.S.); has.ope@tmd.ac.jp (M.H.); hatayama.ope@tmd.ac.jp (T.H.); tagami.ope@tmd.ac.jp (J.T.); nakajima.ope@tmd.ac.jp (M.N.); 2 Institute of Dental Medicine, First Faculty of Medicine of the Charles University and General University Hospital in Prague, Karlovo namesti 32, 121-11 Prague, Czech Republic; 3 Department of Regenerative Dental Medicine, Tokushima University Graduate School of Biomedical Sciences, 3-18-15, Kuramotocho, Tokushima 770-8504, Japan; k.yonekura@tokushima-u.ac.jp; 4 Department of Oral Prosthetic Engineering, Graduate School of Medical and Dental Sciences, Tokyo Medical and Dental University, 1-5-45 Yushima, Bunkyo-ku, Tokyo 113-8510, Japan; ikedsdt@tmd.ac.jp

**Keywords:** antioxidant, dental adhesives, sodium hypochlorite, tooth erosion

## Abstract

The effects of deproteinization using sodium hypochlorite (NaOCl) and the subsequent application of an antioxidant (sodium *p*-toluenesulfinate, STS) onto the bonding durability of universal adhesives on eroded dentin were investigated. Untreated sound dentin served as the control, whereas eroded dentin, which had been prepared by pH-cycling in 1% citric acid and a remineralization solution, was either untreated, deproteinized with a 10% NaOCl gel or deproteinized with the 10% NaOCl gel and subsequently treated with an STS-containing agent. The dentin surfaces were bonded using a universal adhesive (Clearfil Universal Bond Quick, Scotchbond Universal or G-Premio Bond), and the micro-tensile bond strength (µTBS) test was performed after 24 h or 10,000 thermal cycles. The µTBS data were statistically analyzed using a three-way ANOVA and Tukey’s HSD post hoc tests. The lowest µTBS was measured on untreated eroded dentin (*p* < 0.001). Deproteinization of eroded dentin resulted in µTBS similar to untreated sound dentin (*p* > 0.05), but the highest µTBS was obtained if deproteinization was followed by the application of STS. Thermocycling significantly decreased µTBS in all groups (*p* < 0.001), except for STS-treated deproteinized eroded dentin (*p* > 0.05). This indicated that deproteinization, followed by the application of STS, could enhance the bonding durability of universal adhesives on eroded dentin.

## 1. Introduction

While the incidence of caries in developed countries tends to decrease, dental erosion becomes increasingly frequent [1]. The prevalence of dental erosion in permanent dentition is currently estimated to be 20–45% [1], and this is even more prevalent in risk groups, such as patients with gastroesophageal reflux disease, eating disorders, special diets and those who frequently consume acidic beverages, drugs or alcohol [1,2,3,4]. Dental erosion manifests as erosive tooth wear, i.e., non-carious loss of hard dental tissues caused by their demineralization, which leads to a decrease in their mechanical properties and increased susceptibility to abrasion or attrition [5]. Initially, only the enamel is affected, but long-term exposure to acids results in the involvement of dentin, which is less resistant to demineralization [6]. Consequently, the loss of dental tissues is accelerated, and restorative treatment is required to reduce hypersensitivity and prevent further progression, leading to the inflammation of the pulp [5,7].

According to the European consensus statement on managing severe tooth wear, minimally invasive treatment options are recommended, such as direct composite restorations or partial crowns [8]. However, these restorations rely on adhesion, which may be compromised on eroded dentin [9,10] because a thick layer of exposed collagen fibrils is present on the demineralized surface, interfering with the penetration of monomers contained in dental adhesives [11]. As a result, defects are present in the hybrid layer, and the bond strength may be lower than on sound dentin [9,10]. Moreover, the insufficient penetration of the collagen mesh results in nanoleakage, and the exposed collagen becomes prone to degradation by enzymes such as matrix metalloproteinases (MMPs) [12]. The decomposition of the collagen may contribute to the decreased durability of bonding to eroded dentin, as reported by numerous studies [9,10].

The available evidence was mainly obtained through in vitro studies, where erosion was simulated using various pH-cycling protocols. However, the lack of standardization complicates the interpretation of the results [9,10]. In previous studies, demineralization was most commonly performed using citric acid of various concentrations or soft drinks such as Coca-Cola and Sprite (The Coca-Cola Company, Atlanta, GA, USA). Based on the consensus of the Workshop on Methodology in Erosion Research held in 2010, 1% citric acid should be preferred, as it is more reproducible and controllable than soft drinks, while having a pH similar to that of orange juice [13]. The consensus also stated that the erosive challenge should not exceed a few minutes, to be comparable with real-life situations. For remineralization solutions, artificial saliva was recommended [13] and used in most previous studies, even though the composition was not standardized.

Various mechanical and chemical surface treatments were tested to improve the adhesion to eroded dentin. Mechanical treatments removed the superficial demineralized layer and exposed the underlying sound dentin. As a result, increased bond strength was reported after superficial preparation using a fine diamond bur [11,14] or laser [15]. Chemical treatments were non-invasive, i.e., the superficial dentin was modified rather than removed. Inhibitors of MMPs such as 2% chlorhexidine or benzalkonium chloride were investigated in several studies, as they could prevent the enzymatic degradation of collagen and thus improve the bonding durability, however the results were conflicting [9,14,16,17,18]. On the other hand, deproteinization using sodium hypochlorite (NaOCl) for 40–60 s was found to improve durability [14,19,20], as this can remove the superficial collagen and, therefore, improve the monomer penetration. However, previous reports have shown that the treatment of dentin with NaOCl harms the polymerization of adhesives, as the oxidizing effect leads to premature chain termination [21] and hampers the bonding performance [22]. The effect is dependent on the application time of NaOCl [23] and to counteract this, the application of an antioxidant, e.g., sodium ascorbate, sodium thiosulfate, rosmarinic acid or sodium *p*-toluenesulfinate (STS), was shown to be effective in several studies [21,24,25,26,27]. Moreover, a recent study showed that the application of agents containing sulfinates improved the adhesives’ degree of conversion even on untreated sound dentin [28].

Given the benefits of antioxidants on deproteinized sound dentin, the purpose of this study was to investigate whether the application of STS can improve the bonding to deproteinized eroded dentin. As the combined pretreatment with NaOCl and STS is relatively time-consuming, easy-to-use universal adhesives were employed in this study so that the procedure was not excessively technique-sensitive. In addition, universal adhesives are increasingly popular among clinicians, and recent studies suggest that their durability may have improved [29,30,31,32]. The null hypothesis tested in this study was that the deproteinization using NaOCl and application of STS would not affect the bonding performance of universal adhesives to eroded dentin.

## 2. Materials and Methods

One hundred and twenty-four extracted sound human molars were used in this study, following protocol number D2013-22, approved by the Human Research Ethics Committee of Tokyo Medical and Dental University. They were stored in a periodically exchanged 0.1% thymol solution at 4 °C and used within 6 months of extraction. Their occlusal enamel was removed using a model trimmer, and the exposed mid-coronal dentin surfaces were ground with a wet 320-grit SiC paper (DCCS, Sankyo Fuji Star, Saitama, Japan) for 30 s to create a standardized smear layer. A quarter of the teeth served as a control group, while the rest were subjected to pH-cycling, according to Zimmerli et al. [11]. In brief, the teeth with exposed dentin surfaces were immersed in 1% citric acid (pH = 3.5; Wako Pure Chemical Industries, Osaka, Japan) for 5 min, rinsed with distilled water, and immersed in a remineralization solution with pH adjusted to 6.4 (Table 1) for 3.5 h. The pH-cycling was conducted for 8 consecutive days with 6 cycles per day. The pH of the demineralization and remineralization solution was periodically monitored with a pH meter.

For the micro-tensile bond strength (µTBS) test, 120 teeth were divided into four groups as follows: (1) untreated sound dentin (control group), (2) untreated eroded dentin, (3) eroded dentin deproteinized using a 10% NaOCl gel (AD gel; Kuraray Noritake Dental, Tokyo, Japan) and (4) eroded dentin deproteinized using the 10% NaOCl gel followed by the application of an STS-containing agent (Accel; Sun Medical, Kyoto, Japan). AD gel was applied with rubbing motion for 60 s, rinsed off with water for 15 s, and the surfaces were mildly air-blown for 10 s. Accel was applied using a disposable microbrush for 10 s and gently air-dried for 10 s. The dentin surfaces were further divided into three subgroups and bonded using the universal adhesives Clearfil Universal Bond Quick (UBQ; Kuraray Noritake Dental, Tokyo, Japan), Scotchbond Universal (SBU; 3M, St. Paul, MN, USA) or G-Premio Bond (GPB, GC, Tokyo, Japan). These universal adhesives were then used in the self-etching mode according to the manufacturers’ instructions, which are listed in Table 2 along with the composition of the materials used. Light-curing was performed using an LED light-curing unit (Valo; Ultradent Products, South Jordan, UT, USA) for 10 s at 1000 mWcm^−2^. The bonded surfaces were built up with a hybrid resin composite (Clearfil AP-X, shade A2; Kuraray Noritake Dental, Tokyo, Japan) to a height of 6 mm in three increments. Each increment was light-cured for 40 s with the Valo light-curing unit.

After storage in distilled water at 37 °C for 24 h, the specimens were sectioned in two directions perpendicular to the adhesive layer into beams (cross-sectional area: 1.0 × 1.0 mm) using a low-speed diamond saw (Isomet 1000; Buehler, Lake Bluff, IL, USA) under water cooling. The beams’ dimensions were measured using a digital calliper (Mitutoyo CD15; Mitutoyo, Kawasaki, Japan) for precise bonding area calculation. Four beams from the central area of each specimen were used for the testing. Half of the beams were tested immediately, while the other half underwent artificial aging simulated by 10,000 thermal cycles between 5 °C and 55 °C (dwell time 30 s, transfer time 5 s). After the respective storage condition, the beams were individually glued to a µTBS testing jig using a cyanoacrylate adhesive (Model Repair II Blue; Dentsply Sirona, Charlotte, NC, USA), mounted in a tabletop testing machine (EZ-SX; Shimadzu, Kyoto, Japan), and subjected to the µTBS test at a crosshead speed of 1.0 mm/min.

The fractured specimens were mounted on brass stubs, desiccated for 24 h, sputter-coated with gold, and their failure mode was determined using a scanning electron microscope (SEM; JSM-5310; JEOL, Tokyo, Japan) at 75× magnification. The failure modes were classified as: (1) a cohesive failure in dentin (70–100% of the failure occurred in dentin), (2) a dentin/adhesive interfacial failure (70–100% of the failure occurred at the interface between dentin and the adhesive), (3) an adhesive/composite interfacial failure (70–100% of the failure occurred at the interface between the resin composite and adhesive), (4) a cohesive failure in the resin composite (70–100% of the failure occurred in the resin composite build-up) or (5) a mixed failure (at least two of the failure patterns mentioned above were observed, but none of them covered over 70%). In addition, SEM was used to observe morphological changes on the dentin surface in the experimental groups. The remaining four teeth (one sound and three eroded) were used and pretreated as described above. Initially, the specimens were fixed in 2.5% glutaraldehyde in 0.1 M sodium cacodylate (Na(CH_3_)_2_AsO_2_) buffer at pH 7.4 for 12 h at 48 °C and rinsed with 20 mL of 0.2 M sodium cacodylate buffer at pH 7.4 for 1 h. The specimens were then dehydrated in ascending grades of ethanol: 25% (20 min), 50% (20 min), 75% (20 min), 95% (30 min) and 100% (60 min), followed by immersion in hexamethyldisilazane for 10 min [33]. After sputter-coating with gold, the dentin surfaces were observed at 5000x magnification.

Statistical analyses were performed using the SPSS Statistics software (version 27.0 for Windows; IBM, Armonk, NY, USA) at the significance level of 0.05. For the µTBS data, the tooth was considered a statistical unit (*n* = 5), i.e., µTBS values of the four beams originating from each tooth were averaged, and the mean was processed statistically. Since the Shapiro–Wilk test indicated that µTBS data were normally distributed and Levene’s test confirmed the homogeneity of variances, the results were analyzed with a three-way ANOVA (variables: dentin pretreatment, adhesive, aging). Pairwise comparisons were performed using Tukey’s HSD post hoc tests. Failure mode distributions were analyzed using Fisher’s exact test.

## 3. Results

### 3.1. µTBS

The µTBS results are presented in Table 3. The three-way ANOVA revealed that the effects of dentin pretreatment (*p* < 0.001), aging (*p* < 0.001) and adhesive (*p* = 0.006) were significant. The interaction between dentin pretreatment and aging was significant as well (*p* = 0.003), whereas interactions between dentin pretreatment and material (*p* = 0.908), and between material and aging (*p* = 0.448) were not significant. The three-way interaction was not significant either (*p* = 0.869). Regardless of the adhesive and aging, µTBS to eroded dentin was significantly lower compared to other groups (*p* < 0.001). µTBS to sound dentin (control group) was similar to deproteinized eroded dentin, yet decreased significantly after thermocycling (*p* < 0.001). The application of the STS-containing agent increased µTBS with all adhesives, but the difference was not significant with GPB (*p* > 0.05). Thermocycling had no significant effect on µTBS to deproteinized eroded dentin treated with the STS-containing agent (*p* > 0.05). Among the adhesives, the µTBS of UBQ was significantly higher compared to SBU (*p* = 0.049) and GPB (*p* = 0.006), which did not significantly differ from each other (*p* = 0.736).

### 3.2. Failure Mode Analysis

**Figure 1:**
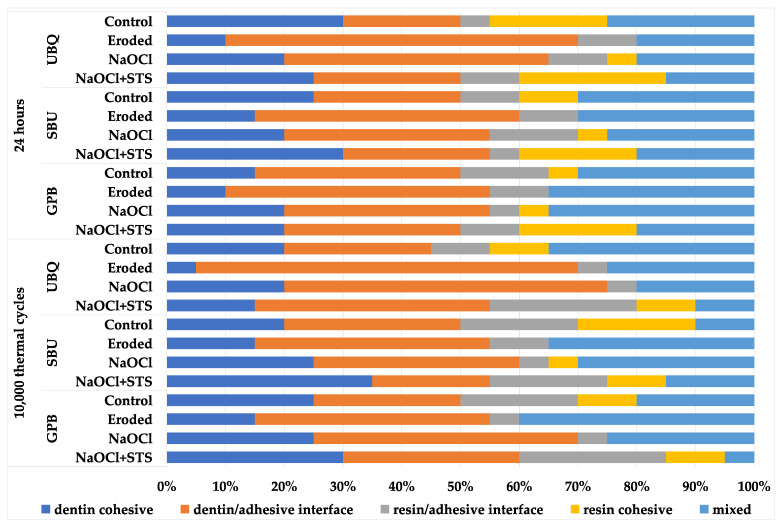
Distribution of failure modes in percentage.

The failure mode distributions are shown in Figure 1. There were no significant differences between the experimental groups (*p* > 0.05). However, slightly more dentin/adhesive interfacial failures were observed on untreated eroded dentin, whereas cohesive failures in dentin and the resin composite tended to be more frequent on deproteinized eroded dentin treated with the STS-containing agent. No pre-testing failures occurred.

### 3.3. Morphological Analysis of the Dentin Surfaces

**Figure 2:**
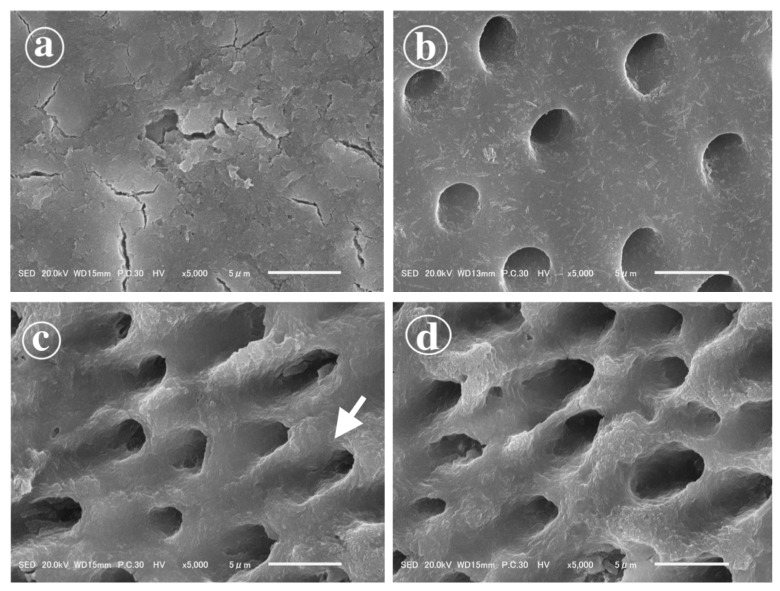
Scanning electron micrographs of the dentin surfaces. (**a**) The dentin surface in the control group was covered by a smear layer. (**b**) Smear layer was not present on eroded dentin, and collagen fibrils were observed on the untreated surface. (**c**) After deproteinization of eroded dentin with 10% NaOCl, the orifices of dentinal tubules were enlarged to a funnel shape (indicated by the white arrow), and the surface was very porous. (**d**) The application of the STS-containing agent did not alter the surface morphology.

Representative SEM images of the dentin surfaces are presented in Figure 2. In the control group (Figure 2a), the dentin surface was covered with a compact smear layer and dentinal tubules were obliterated. pH-cycling removed the smear layer and exposed the orifices of dentinal tubules (Figure 2b–d). On the untreated eroded dentin, collagen fibrils were observed (Figure 2b); however, deproteinization with 10% NaOCl removed them (Figure 2c,d). The deproteinized surfaces were very porous, and dentinal tubules’ orifices were enlarged to a funnel shape (Figure 2c,d). The application of the STS-containing agent did not significantly alter the surface morphology of the deproteinized eroded dentin.

## 4. Discussion

While the achievement of durable adhesion on sound dentin is challenging, bonding to eroded dentin is even more complex as the cyclic demineralization exposes a layer of disorganized collagen network on the eroded dentin surface [6] (Figure 2b). As a result, the penetration of adhesives into the underlying dentin is hindered, and a defective hybrid layer is formed, leading to lower bond strengths and reduced durability [9,10]. This was confirmed by the present study, as bonding to untreated eroded dentin resulted in the lowest 24 h and aged µTBS, and dentin/adhesive interfacial failures prevailed. However, the objective of this study was to examine the effect of a deproteinizing pretreatment using a 10% NaOCl gel and the subsequent application of an STS-containing agent on the bonding performance of universal adhesives to eroded dentin. As deproteinization significantly increased µTBS and µTBS after the application of STS surpassed that of untreated sound dentin, the null hypothesis was rejected.

NaOCl is a strongly alkaline, non-specific proteolytic compound that can decompose the exposed collagen layer on eroded dentin. The deproteinizing ability of NaOCl was confirmed using SEM (Figure 2c,d), which revealed that collagen was removed, funnel-shaped orifices of dentinal tubules were exposed, and the surface was more porous compared to untreated sound or eroded dentin (Figure 2a,b). These changes presumably facilitated the penetration of the adhesives and hybridization, thus leading to a significant increase in µTBS after deproteinization. There was also no significant difference between µTBS to deproteinized eroded dentin and the control group. These findings agree with a study by Deari et al., in which a similar deproteinizing pretreatment was used [14]. However, Deari et al. did not measure aged bond strength, which highlights a limitation of their study, as the correlation of immediate bond strength with clinical outcomes is limited [34]. Augusto et al. used 10% NaOCl for 60 s and revealed that deproteinization significantly improved µTBS to eroded dentin [19]. Furthermore, µTBS to deproteinized eroded dentin was not significantly affected by 5000 thermal cycles, and it was significantly higher than the control group after aging, regardless of etching mode [19]. Siqueira et al. used a 5.2% solution of NaOCl for 40 s and found that the µTBS of a self-etching adhesive to deproteinized eroded dentin exceeded µTBS to sound dentin both immediately and after 3 years of water storage [20]. It was also found that the deproteinization of eroded dentin significantly decreased nanoleakage [20], and therefore contributed to excellent bonding durability. In this study, µTBS to deproteinized dentin significantly decreased after 10,000 thermal cycles. We speculate that the differences might influence this in pH-cycling protocols and the adhesive systems used.

As a part of the deproteinizing action of NaOCl, free radicals were formed, and they interfered with the propagation of vinyl radicals, which in turn resulted in premature chain termination and the incomplete polymerization of adhesives [21,24,25,26,27]. This was the rationale for using STS, as previous studies showed that applying antioxidants or reducing agents could improve the bond strength to NaOCl-deproteinized dentin [21,24,25,26,27]. Furthermore, sulfinates could substitute tertiary amines as co-initiators of camphorquinone [35], which is used as a photoinitiator in most adhesive systems, and sulfinate-containing agents were recently reported to increase the degree of conversion of light-cured adhesives on dentin [28]. In this study, the treatment of deproteinized eroded dentin with the STS-containing agent resulted in the highest µTBS. Moreover, the bond strength was not significantly affected by thermocycling, as opposed to other tested groups, including sound dentin. Our assumption is that STS improved the degree of conversion of the tested adhesives [28], thus contributing to the improved µTBS, particularly after thermocycling [31]. Notably, while the bonding durability was improved, this was at the expense of two additional steps, which made the bonding procedure more elaborate and time-consuming. As such, a slight preparation of the eroded dentin using a diamond bur [11,14], followed by the standard application of any adhesive may be preferred by some clinicians, even though such an approach is slightly more invasive.

In the control group, µTBS was similar to deproteinized eroded dentin, but the characteristics of the bonding surface were dissimilar. On eroded dentin, the smear layer was removed by pH-cycling (Figure 2c), and deproteinization created a mineral-rich porous surface suitable for the formation of micromechanical and chemical bonding. In contrast, the dentin surface in the control group was entirely covered with a compact smear layer (Figure 2a), the presence of which could hinder ideal bonding as the smear layer interferes with monomer infiltration and the chemical interaction of the adhesive monomers with the underlying intact dentin [36,37]. Additionally, the formation of the hybridized smear layer above the authentic hybrid layer [38] was thought to have an adverse effect on bonding durability [37]. While these factors could have harmed immediate and aged µTBS, they were probably balanced by sound dentin acting as a better substrate for bonding, and the absence of radicals, which could impede the polymerization of adhesives on deproteinized dentin.

While the effect of dentin pretreatment was similar for all the tested adhesives, their µTBS differed, as UBQ exhibited significantly higher values than SBU and GPB. Even though all the adhesives contained 10-methacryloyloxydecyl dihydrogen phosphate (10-MDP), impurities were found to adversely affect hybridization, the formation of calcium salts, and nanolayering [39]. Since Kuraray Noritake Dental reportedly produced the purest 10-MDP [39], this could have contributed to the high µTBS of UBQ. UBQ also contains novel hydrophilic amide monomers [30], allowing for the reduced content of 2-hydroxyethyl methacrylate (HEMA). This may support the durability of UBQ, as HEMA was associated with increased water sorption and a decreased degree of conversion [40,41]. However, the HEMA-free adhesive GPB did not exhibit superior durability. This may be attributed to the content of 4-methacryloxyethyl trimellitic acid, whose bonding potential is lower compared to 10-MDP [42]. In addition, the use of acetone as a solvent in GPB might be suboptimal on eroded dentin since its ability to re-expand collapsed collagen fibers is limited due to its low hydrogen-bonding capacity [43]. On the other hand, GPB was the only tested adhesive not affected by the application of STS, which might indicate that its polymerization was not impaired by the presence of radicals produced by NaOCl. This could be due to the content of diphenyl(2,4,6-trimethylbenzoyl)phosphine oxide known as TPO, a very efficient photoinitiator, which does not require tertiary amines as co-initiators [31].

The main limitation of this study is that it was performed in vitro on artificially eroded dentin surfaces. While the pH-cycling models attempted to mimic the process of tooth erosion by cyclic demineralization and remineralization, the intraoral conditions are much more complex. Firstly, the collagen network on eroded surfaces may have been decomposed by various enzymes, or as a result of chewing and the brushing of teeth. Secondly, the in vivo process of erosion is much longer compared to pH-cycling, and provided the eroded teeth are vital, the structure of dentin may have changed in reaction to the cyclic acid attacks. Thirdly, the bonding surfaces involve both dentin and enamel, but in vitro studies usually investigate these substrates separately. Therefore, their outcomes should be interpreted with caution, and they should be confirmed by in vivo studies.

## 5. Conclusions

Within the limitations of this in vitro study, we concluded that deproteinization with NaOCl significantly increased the bond strength of universal adhesives to eroded dentin. When combined with the subsequent application of a sodium *p*-toluenesulfinate-containing agent, the bond strength and durability were even superior to sound dentin.

## Figures and Tables

**Table 1:** Composition of the demineralization and remineralization solution (pH-cycling).

Solution	Composition
Demineralization	1% citric acid (pH = 3.5)
Remineralization	0.002 g ascorbic acid, 0.58 g NaCl, 0.17 g CaCl_2_, 0.16 g NH_4_Cl, 1.27 g KCl, 0.16 g NaSCN, 0.33 g KH_2_PO_4_, 0.34 g Na_2_HPO_4_ dissolved in 1 L of demineralized water (pH adjusted to 6.4 with HCl)

**Table 2:** Composition and application procedure of materials used in this study.

Material (Manufacturer)	Composition	pH	Application Procedure
Clearfil Universal Bond Quick (UBQ; Kuraray Noritake Dental, Tokyo, Japan)	10-MDP, Bis-GMA, HEMA, hydrophilic amide monomers, colloidal silica, coupling agent, sodium fluoride, CQ, ethanol, water	2.3	Apply to dentin with rubbing motion Dry with mild air pressure for 5 s Light cure for 10 s
Scotchbond Universal Adhesive (SBU; 3M, St.Paul, MN, USA)	10-MDP, dimethacrylate resins, Bis-GMA, HEMA, Vitrebond copolymer, silane, ethanol, water, filler, initiator	2.7	Apply to dentin with rubbing motion for 20 s Dry with mild air pressure for 5 s Light cure for 10 s
G-Premio Bond (GPB; GC, Tokyo, Japan)	10-MDP, 4-MET, MEPS, methacrylate monomer, acetone, water, initiator, silica	1.5	Apply to dentin with rubbing motion for 10 s Dry with strong air pressure for 5 s Light cure for 10 s
Accel (Sun Medical, Kyoto, Japan)	sodium *p*-toluenesulfinate, ethanol, water		Apply to dentin for 10 s Dry with gentle air pressure for 10 s
AD gel (Kuraray Noritake Dental, Tokyo, Japan)	10% sodium hypochlorite, thickener		Apply to dentin with rubbing motion for 60 s Rinse off with water for 15 s Dry with mild air pressure for 10 s
Clearfil AP-X (shade A2; Kuraray Noritake Dental, Tokyo, Japan)	Bis-GMA, TEGDMA, silanated barium glass filler, silanated silica filler, silanated colloidal silica, CQ, initiators, accelerators, pigments		Place AP-X onto the bonded dentin surface in increments of 2 mm thickness Light cure each increment for 40 s

Abbreviations: 10-MDP: 10-methacryloyloxydecyl dihydrogen phosphate; Bis-GMA: bisphenol A diglycidylmethacrylate; HEMA: 2-hydroxyethyl methacrylate; CQ: camphorquinone; 4-MET: 4-methacryloxyethyl trimellitic acid; MEPS: methacryloyloxyalkyl thiophosphate methylmethacrylate; TEGDMA: triethyleneglycol dimethacrylate.

**Table 3:** µTBS results: Mean (SD) in MPa.

Adhesives	Dentin Pretreatment	24 h	10,000 Thermal Cycles
UBQ	Control	63.1 (10.7) ^A,b^	50.1 (7.6) ^B,b^
	Eroded	49.4 (9.7) ^A,c^	22.1 (5.7) ^B,c^
	NaOCl	61.0 (8.5) ^A,b^	44.3 (8.3) ^B,b^
	NaOCl + STS	71.0 (9.3) ^A,a^	64.5 (9.8) ^A,a^
SBU	Control	57.6 (6.7) ^A,ab^	47.0 (5.8) ^B,ab^
	Eroded	39.2 (8.7) ^A,c^	23.5 (5.8) ^B,c^
	NaOCl	55.9 (11.9) ^A,b^	43.8 (7.1) ^B,b^
	NaOCl + STS	63.9 (7.6) ^A,a^	58.2 (11.7) ^A,a^
GPB	Control	53.1 (9.3) ^A,b^	42.9 (7.2) ^B,b^
	Eroded	40.4 (8.5) ^A,c^	20.4 (4.1) ^B,c^
	NaOCl	60.9 (8.7) ^A,ab^	40.1 (10.2) ^B,ab^
	NaOCl + STS	61.3 (8.8) ^A,a^	58.7 (8.2) ^A,a^

Different uppercase letters indicate a statistically significant difference between aging conditions (in rows). Different lowercase letters indicate a statistically significant difference between dentin pretreatments (in columns) for each adhesive.

## Data Availability

Data available on request from the corresponding author.
